# Excitatory deep brain stimulation quenches beta oscillations arising in a computational model of the subthalamo-pallidal loop

**DOI:** 10.1038/s41598-022-10084-4

**Published:** 2022-05-12

**Authors:** Seyed Mojtaba Alavi, Amin Mirzaei, Alireza Valizadeh, Reza Ebrahimpour

**Affiliations:** 1 Faculty of Computer Engineering, Shahid Rajaee Teacher Training University, Tehran, Iran; 2 School of Cognitive Sciences (SCS), Institute for Research in Fundamental Sciences (IPM), Tehran, Iran; 3 Insilico Biotechnology AG, Stuttgart, Germany; 4 Department of Physics, Institute for Advance Studies in Basic Sciences (IASBS), Zanjan, Iran; 5 School of Biological Sciences, Institute for Research in Fundamental Sciences (IPM), Tehran, Iran

**Keywords:** Biophysical models, Dynamical systems, Network models, Neural decoding

## Abstract

Parkinson’s disease (PD) is associated with abnormal $\beta$ band oscillations (13–30 Hz) in the cortico-basal ganglia circuits. Abnormally increased striato-pallidal inhibition and strengthening the synaptic coupling between subthalamic nucleus (STN) and globus pallidus externa (GPe), due to the loss of dopamine, are considered as the potential sources of $\beta$ oscillations in the basal ganglia. Deep brain stimulation (DBS) of the basal ganglia subregions is known as a way to reduce the pathological $\beta$ oscillations and motor deficits related to PD. Despite the success of the DBS, its underlying mechanism is poorly understood and, there is controversy about the inhibitory or excitatory role of the DBS in the literature. Here, we utilized a computational network model of basal ganglia which consists of STN, GPe, globus pallidus interna, and thalamic neuronal population. This model can reproduce healthy and pathological $\beta$ oscillations similar to what has been observed in experimental studies. Using this model, we investigated the effect of DBS to understand whether its effect is excitatory or inhibitory. Our results show that the excitatory DBS is able to quench the pathological synchrony and $\beta$ oscillations, while, applying inhibitory DBS failed to quench the PD signs. In light of simulation results, we conclude that the effect of the DBS on its target is excitatory.

## Introduction

Parkinson’s disease (PD) results from malfunctioning of basal ganglia (BG)^1–3^. This malfunctioning follows degeneration of dopaminergic neurons in pars compacta section of the substantia nigra (SNc)^2,4^. Rigidity, bradykinesia, tremor, and postural instability are common signs of PD^5,6^. In addition, this disorder is associated with excessive synchronization and abnormally $\beta$ band (13–30 Hz) oscillations in subregions of BG^6–10^ and enhanced $\beta$ oscillations are known as the biomarker of the PD^11–13^. However, the source of the oscillations is still under debate. Several studies have suggested the subthalamo-pallidal circuit as the main source of the generation of the $\beta$ oscillations in experimental and computational studies^7,8,14–24^. Other studies have shown that $\beta$ oscillations can alternatively be induced from to cortex to BG^6,25,26^.

Furthermore, deep brain stimulation (DBS) of the BG subregions, mainly the subthalamic nucleus (STN), is a standard approach to treating PD^27–33^. Although the DBS quenches the $\beta$ oscillations and improves the PD motor symptoms, its underlying mechanism is poorly understood^34–37^ and, there is a controversy between the excitatory or inhibitory effect of DBS on STN.

Reduction of firing rate of the stimulated neuronal area has been observed in human^38,39^ and monkeys^40^ with PD which remarks the inhibitory role of the DBS. Several mechanisms are suggested to explain the inhibitory role of the DBS such as depolarization block^36,41^, inactivation of voltage-gated currents^42–44^, and activation of inhibitory afferents^38,40,45–50^. In line with these results it is shown that the GPi neurons are inhibited during deep stimulation^45^. On the other hand, the excitatory role of the DBS has also been suggested by several studies^48,51^. Applying DBS on the internal segment of globus pallidus (GPi) reduces firing rates of thalamic neurons which are inhibited by the GPi^52^. Also, applying the DBS on STN neurons (the excitatory neuronal population) increases the firing rate of GPi, globus pallidus externa (GPe), and substantia nigra pars reticulata (SNr) of human and animal with PD^53–55^ which support the excitatory effect of the DBS. In fact, due to the limitations in acquiring and interpretation of electrophysiological data, computational studies can help to unravel the mechanisms through which high-frequency brain stimulation affects the dynamics of the brain circuits in PD.

Computational studies have also explored the effect(s) of the DBS with inhibitory and excitatory pulses on the models of BG with PD signs. In^56^ the BG has been modelled by leaky integrate and fire (LIF) neurons which can generate $\beta$ oscillations in PD condition, and it is shown that the PD $\beta$ oscillations quenched when the model exposed to the inhibitory DBS. While, other computational studies which are based on Hodgkin-Huxley type neurons, used excitatory DBS to suppress PD like oscillations^18,57–62^. However, in these latter studies the PD condition were not characterized by $\beta$ oscillations and consequently the effects of DBS on the oscillatory activity of BG nuclei were not inspected. To investigate whether the effect of the DBS on its target is inhibitory or excitatory, in the current study, we used a computational model based on a variation of the model proposed in^18^ that resulted in $\beta$ oscillations in neuronal populations in PD condition. We assumed that the subthalamo-pallidal circuit generates the pathological $\beta$ oscillations, and we did not inspected the results in the models where other circuits have been introduced as the source of beta oscillations^63–65^. The aim of this study is to investigate whether the role of the DBS on its target is excitatory or inhibitory. Moreover, we investigate how the DBS quenches the abnormal $\beta$ oscillations related to PD in detail. To this end we explained the mechanisms of the $\beta$ rhythm generation and, we found that the excitatory DBS can quench the PD signs and the inhibitory DBS fails to do so.

In this study, we characterized the intensity of $\beta$ oscillations with oscillation index, and neural synchronization with Fano factor, as were defined in a previous study^56^. Since the DBS in that study was inhibitory (in contrary to this study), these analyses are suitable for comparison. Besides, we explored the response of thalamic neurons to cortical input that represents the thalamic fidelity which has investigated experimentally in^66^ and computationally in^67^ to more confirmation of our results.

Despite the $\beta$ rhythm, the resting state tremor is another sign of PD that directly related to thalamic activity^68,69^. Hence, we studied the thalamic activity in the model during the PD condition and the mechanisms of its generation. Our network model showed the excessive thalamic activity during resting state in PD condition. We guess this activity is related to the Tremor. As the DBS has a therapeutic effect on resting-state tremor^35^, we also investigated the effects of DBS in our network model. We found that the excitatory DBS can reduce the excessive thalamic activity while the inhibitory DBS cannot.

## Materials and methods

### Structure of the network model

**Figure 1:**
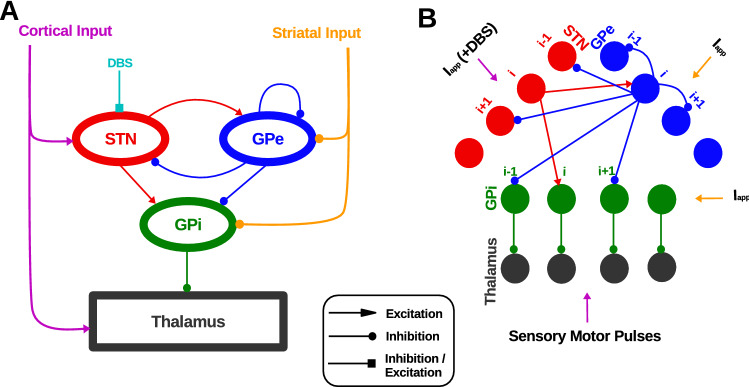
*Network model structure and finding appropriate DBS currents.* (**A**) Schematic of the network model. The DBS is considered to be either inhibitory or excitatory input to the STN. (**B**) Details of the network connectivity. The *i*th STN neuron excites the *i*th GPe and GPi neurons. The *i*th GPe neuron inhibits the $(i-1)$th and $(i+1)$th STN, GPi, and GPe neurons. Each GPi neuron inhibits its corresponding thalamic neuron.

The network model consists of STN, GPe, GPi, and thalamus. Each neuronal population includes 20 Hodgkin-Huxley type neurons. The basic network model structure is similar to^18^ and^17^. the network model structure resembles the sparse pattern of connectivity^70^ proposed in^17^. STN excites GPe and GPi and receives inhibitory input from GPe. Similar to^18^ each STN neuron receives inhibitory input from two GPe neurons. In^18^ each GPe neuron was receiving excitatory input from three STN neurons, while in our simulation, each GPe neuron receives excitatory input from one STN neuron. Similar to^18^ each GPi neuron receives excitatory input from one STN neuron. In addition, each GPi neuron receives inhibitory input from two GPe neurons. In our simulation, each thalamic neuron receives inhibitory input from one GPi neuron, while in^18^ each thalamic neuron was receiving inhibitory input from eight GPi neurons. See Fig. 1 for more details of the network connectivity and structure.

### Neuron and synapse model

The membrane potential of the STN, GPe, and GPi neurons in the network model was computed using the following differential equations:

1 $$\begin{aligned}&\mathrm {C_m v^{\prime }} = -I_L - I_{Na} - I_K - I_{Ca} - I_T - I_{AHP} - I_{pre -> post} + I_{app}, \end{aligned}$$

2 $$\begin{aligned}&\mathrm {X^{\prime }} = \frac{\phi _{_X} (X_{\infty }(v)-X)}{\tau _{_X}(v)}, \end{aligned}$$

where $I_L$, $I_{Na}$, $I_K$, $I_{Ca}$, $I_T$, and $I_{AHP}$ are the leak, sodium, potassium, high threshold calcium, low threshold calcium, and after hyper polarization currents, respectively. $I_{app}$ is the external current applied to the neurons (i.e., the DBS current). $I_{pre->post}$ is synaptic current from the presynaptic to the postsynaptic neuron. *X* represents gating channels such as potassium channels (n), opening (m) and closing (h) sodium channels, and low threshold calcium channels (r). The $\tau _{_X}(v)$ in Eq. (2) is defined as follows:

3 $$\begin{aligned}&\mathrm {\tau _{_X}(v)} = \tau ^0_{_X} + \frac{\tau ^1_{_X}}{1+e^{-(v-\theta ^{\tau }_{_X})}}. \end{aligned}$$

While, in the GPe and GPi neurons the $\tau _{_X}(v)$ is constant and equal to $\tau _r$. The ionic currents used in Eq. (1) were computed as follows:

4 $$\begin{aligned}&\mathrm {I_L} = g_{_L} (v-E_L), \end{aligned}$$

5 $$\begin{aligned}&\mathrm {I_{Na}} = g_{_{Na}} m^3_{\infty }(v) h (v-E_{Na}), \end{aligned}$$

6 $$\begin{aligned}&\mathrm {I_K} = g_{_K} n^4 (v- E_K), \end{aligned}$$

7 $$\begin{aligned}&\mathrm {I_{Ca}} = g_{_{Ca}} s^2_{\infty }(v)(v-E_{Ca}), \end{aligned}$$

8 $$\begin{aligned}&\mathrm {I_T} = g_{_T} a^3_{\infty }(v) b^2_{\infty }(r)(v-E_{Ca}). \end{aligned}$$

In the Eqs. (4) to (8) the $X = n, h$ is the ionic gating channel variables (h for closing sodium channel and n for potassium). In these equations, the $X_{\infty } = m, a, r$ or *s* is the steady-state of the ionic gating channels (m for opening sodium channel, a for T-type and s for L-type calcium channel) and is computed by the Eq. (9).

9 $$\begin{aligned}&\mathrm {X_{\infty }(v)} = \frac{1}{1+e^{-(v-\theta _X)/\sigma _X}}, \end{aligned}$$

But, the function $b_{\infty }(r)$ used in 8 is computed with different equation:

10 $$\begin{aligned}&\mathrm {b_{\infty }(r)} = \frac{1}{1+e^{(r-\theta _b)/\sigma _b}} - \frac{1}{e^{-\theta _b/\sigma _b}}. \end{aligned}$$

The after hyper-polarization (AHP) current used in Eq. (1) ($I_{AHP}$) is

11 $$\begin{aligned} \mathrm {I_{AHP}}= g_{_{AHP}}(v-E_K)([Ca]/([Ca]+k_1)), \end{aligned}$$

where the [*Ca*] is the intra-cellular calcium concentration:

12 $$\begin{aligned} \mathrm {[Ca]^{'}} = \varepsilon (-I_{Ca}-I_T-k_{Ca}[Ca]). \end{aligned}$$

The parameters and their values of STN, GPe, and GPi neurons are presented in the Tables 1, 2, 3.

**Table 1:** Parameters and their corresponding values of STN neurons.

Parameter	Value	Parameter	Value	Parameter	Value	Parameter	Value
$g_{_L}$	2.25 $nS/\mu m^2$	$\tau ^0_h$	1 ms	$\theta _a$	− 63 mV	$\sigma _n$	8 mV
$g_{_{Na}}$	30 $nS/\mu m^2$	$\tau ^0_n$	1 ms	$\theta _b$	0.4 mV	$\sigma _r$	− 2 mV
$g_{_K}$	40 $nS/\mu m^2$	$\tau ^0_r$	40 ms	$\theta _s$	− 39 mV	$\sigma _a$	7.8 mV
$g_{_T}$	0.5 $nS/\mu m^2$	$\phi _h$	5	$\theta ^{\tau }_h$	− 57 mV	$\sigma _b$	− 0.1 mV
$g_{_{Ca}}$	0.5 $nS/\mu m^2$	$\phi _n$	5	$\theta ^{\tau }_n$	− 80 mV	$\sigma _s$	8 mV
$g_{_{AHP}}$	9 $nS/\mu m^2$	$\phi _r$	2	$\theta ^{\tau }_r$	68 mV	$\sigma ^{\tau }_h$	− 3 mV
$E_L$	− 60 mV	$k_1$	15	$\theta ^H$	− 39 mV	$\theta _X{\tau }_n$	− 26 mV
$E_{Na}$	55 mV	$k_{Ca}$	22.5	$\theta$	20 mV	$\sigma ^{\tau }_r$	− 2.2 mV
$E_K$	− 80 mV	$\epsilon$	$3 \times 10^{-5} ms^{-1}$	$\alpha$	2 $ms^{-1}$	$\sigma ^H$	8 mV
$E_{Ca}$	140 mV	$\theta _m$	− 30 mV	$g_{_{GPe\rightarrow STN}}$	$2.2 \mapsto 7^*$ $nS/\mu m^2$	$\beta$	0.08 $ms^{-1}$
$\tau ^1_h$	500 ms	$\theta _h$	− 39 mV	$E_{GPe \rightarrow STN}$	− 85 mV	$I_{app}$	$8.4 \mapsto 3^*$ $pA/\mu m^2$
$\tau ^1_h$	100 ms	$\theta _n$	− 32 mV	$\sigma _m$	15 mV	$C_m$	1
$\theta ^1_r$	17.5 mV	$\theta _r$	− 67 mV	$\sigma _h$	− 3.1 mV		

The stars indicate transition from healthy to PD.

**Table 2:** Parameters and their corresponding values of GPe neurons.

Parameter	Value	Parameter	Value	Parameter	Value	Parameter	Value
$g_{_L}$	0.1 $nS/\mu m^2$	$\tau ^0_n$	0.05 ms	$\theta _s$	− 35 mV	$\sigma _r$	− 2 mV
$g_{_{Na}}$	120 $nS/\mu m^2$	$\tau _r$	30 ms	$\theta ^{\tau }_h$	− 40 mV	$\sigma _a$	2 mV
$g_{_K}$	30 $nS/\mu m^2$	$\phi _h$	0.135	$\theta ^{\tau }_n$	− 40 mV	$\sigma _s$	2 mV
$g_{_T}$	0.5 $nS/\mu m^2$	$\phi _n$	0.165	$\theta ^{H}$	− 57 mV	$\sigma ^{\tau }_h$	− 12 mV
$g_{_{Ca}}$	0.15 $nS/\mu m^2$	$\phi _r$	1	$\theta$	30 mV	$\sigma ^{\tau }_n$	− 12 mV
$g_{_{AHP}}$	30 $nS/\mu m^2$	$k_1$	30	$\alpha$	5 $ms^{-1}$	$\sigma ^{H}$	2 mV
$E_L$	− 55 mV	$k_{Ca}$	2.4	$g_{_{STN \rightarrow GPe}}$	$0.01 \mapsto 0.55^*$ $nS/\mu m^2$	$\beta$	0.14 $ms^{-1}$
$E_{Na}$	55 mV	$\epsilon$	0.0055 $ms^{-1}$	$E_{STN \rightarrow GPe}$	0 mV	$I_{app}$	$5.9 \mapsto 0.5^*$ $pA/\mu m^2$
$E_K$	− 80 mV	$\theta _m$	− 37 mV	$g_{_{GPe \rightarrow GPe}}$	$0.01 \mapsto 0.9^*$ $nS/\mu m^2$	$C_m$	1
$E_{Ca}$	120 mV	$\theta _h$	− 58 mV	$E_{_{GPe\rightarrow GPe}}$	− 100 mV		
$\tau ^1_h$	0.27 ms	$\theta _n$	− 50 mV	$\sigma _m$	10 mV		
$\tau ^1_n$	0.27 ms	$\theta _r$	− 70 mV	$\sigma _h$	− 12 mV		
$\theta ^0_h$	0.05 mV	$\theta _a$	− 57 mV	$\sigma _n$	14 mV		

The stars indicate transition from healthy to PD.

**Table 3:** Parameters and their corresponding values of GPi neurons.

Parameter	Value	Parameter	Value	Parameter	Value	Parameter	Value
$g_{_L}$	0.1 $nS/\mu m^2$	$\tau ^0_n$	0.05 ms	$\theta _s$	− 35 mV	$\sigma _r$	− 2 mV
$g_{_{Na}}$	120 $nS/\mu m^2$	$\tau _r$	30 ms	$\theta ^{\tau }_h$	− 40 mV	$\sigma _a$	2 mV
$g_{_K}$	30 $nS/\mu m^2$	$\phi _h$	0.1	$\theta ^{\tau }_n$	− 40 mV	$\sigma _s$	2 mV
$g_{_T}$	0.5 $nS/\mu m^2$	$\phi _n$	0.135	$\theta ^{H}$	− 57 mV	$\sigma ^{\tau }_h$	− 12 mV
$g_{_{Ca}}$	0.15 $nS/\mu m^2$	$\phi _r$	1	$\theta$	30 mV	$\sigma ^{\tau }_n$	− 12 mV
$g_{_{AHP}}$	30 $nS/\mu m^2$	$k_1$	30	$\alpha$	5 $ms^{-1}$	$\sigma ^{H}$	2 mV
$E_L$	− 55 mV	$k_{Ca}$	2.4	$g_{_{STN \rightarrow GPe}}$	$0.005 \mapsto 1.1^*$ $nS/\mu m^2$	$\beta$	0.14 $ms^{-1}$
$E_{Na}$	55 mV	$\epsilon$	0.0055 $ms^{-1}$	$E_{STN \rightarrow GPe}$	0 mV	$I_{app}$	$7.7 \mapsto 4^*$ $pA/\mu m^2$
$E_K$	− 80 mV	$\theta _m$	− 37 mV	$g_{_{GPe \rightarrow GPe}}$	$0.01 \mapsto 1.9^*$ $nS/\mu m^2$	$C_m$	1
$E_{Ca}$	120 mV	$\theta _h$	− 58 mV	$E_{_{GPe\rightarrow GPe}}$	− 100 mV		
$\tau ^1_h$	0.27 ms	$\theta _n$	− 50 mV	$\sigma _m$	10 mV		
$\tau ^1_n$	0.27 ms	$\theta _r$	− 70 mV	$\sigma _h$	− 12 mV		
$\theta ^0_h$	0.05 mV	$\theta _a$	− 57 mV	$\sigma _n$	14 mV		

The stars indicate transition from healthy to PD.

The membrane potential of thalamic neurons in the network model is computed using the following differential equations:

13 $$\begin{aligned}&\mathrm {C_m v^{\prime }} = -I_L - I_{Na} - I_K - I_T - I_{GPi -> Th} + I_{SMC}, \end{aligned}$$

14 $$\begin{aligned}&\mathrm {h^{\prime }} = \frac{h_{\infty }(v)-h}{\tau _h(v)} , \end{aligned}$$

15 $$\begin{aligned}&\mathrm {r^{\prime }} = \frac{r_{\infty }(v) - r}{\tau _r(v)}, \end{aligned}$$

where $I_L$, $I_{Na}$, $I_K$, and $I_T$ are the leak, sodium, potassium, and low threshold calcium currents, respectively. $I_{GPi -> Th}$ is the synaptic current from a GPi neuron to a thalamic neuron in the network model. The $I_{SMC}$ represents cortico-thalamic sensorimotor pulses applied to the thalamic neurons. The Eqs. (16) to (19) compute the ionic currents used in Eq. (13).

16 $$\begin{aligned}&\mathrm {I_L} = g_{_L} (v_{_{Th}} - E_L), \end{aligned}$$

17 $$\begin{aligned}&\mathrm {I_{Na}} = g_{_{Na}} m^3_{\infty }(v_{_{Th}})h_{_{Th}}(v_{_{Th}} - E_{Na}), \end{aligned}$$

18 $$\begin{aligned}&\mathrm {I_K} = g_{_K}[0.75(1-h_{_{Th}})]^4[v_{_{Th}} - E_K], \end{aligned}$$

19 $$\begin{aligned}&\mathrm {I_T} = g_{_T} p^2_{\infty }(v_{_{Th}})r_{_{Th}}(v_{_{Th}} - E_T)m \end{aligned}$$

and the functions used in Eqs. (14) to (19) are computed as follows:

20 $$\begin{aligned}&\mathrm {h_{\infty }(v_{_{Th}})} = \frac{1}{1+e^{(v_{_{Th}}+41)/4}}, \end{aligned}$$

21 $$\begin{aligned}&\mathrm {r_{\infty }(v_{_{Th}})} = \frac{1}{1+e^{(v_{_{Th}}+48)/4}}, \end{aligned}$$

22 $$\begin{aligned}&\mathrm {\tau _h(v_{_{Th}})} = \frac{1}{a_h(v_{_{Th}}) + b_h(v_{_{Th}})}, \end{aligned}$$

23 $$\begin{aligned}&\mathrm {\tau _r (v_{_{Th}})} = 28 + e^{-(v_{_{Th}} + 25)/10.5}, \end{aligned}$$

24 $$\begin{aligned}&\mathrm {m_{\infty }(v_{_{Th}})} = \frac{1}{1 + e^{-(v_{_{Th}} + 37)/7}}, \end{aligned}$$

25 $$\begin{aligned}&\mathrm {p_{\infty }(v_{_{Th}})} = \frac{1}{1 + e^{-(v_{_{Th}} + 60)/6.2}}, \end{aligned}$$

which the $a_h(v_{Th})$ and $b_h(v_{Th})$ are

26 $$\begin{aligned}&\mathrm {a_h(v_{_{Th}})} = 0.128e^{-(v_{_{Th}}+46)/18}, \end{aligned}$$

27 $$\begin{aligned}&\mathrm {b_h(v_{_{Th}})} = \frac{4}{1 + e^{-(v_{_{Th}} + 23)/5}}. \end{aligned}$$

The parameters and their values of thalamic neurons are presented in Table 4.

**Table 4:** Thalamic parameters and the corresponding values.

Parameter	Value
$g_{_L}$	0.05 $nS/\mu m^2$
$E_L$	− 70 mV
$g_{_{Na}}$	3 $nS/\mu m^2$
$E_{Na}$	50 mV
$g_{_T}$	5 $nS/\mu m^2$
$E_T$	0 mV
$g_{_{GPi \rightarrow Th}}$	005 $nS/\mu m^2$
$E_{GPi \rightarrow Th}$	− 85 mV
$\theta ^H$	− 57 mV
$\sigma ^H$	2 mV

In addition to modification of the network structure, we modified the network model parameters compared to the Terman et al.^17^ as follows: for the STN neurons $g_{_{Na}}$ was decreased from 37.5 to $30 \, nS/\mu m^2$. The $g_{_{K}}$ was decreased from 45 to $40 \, nS/\mu m^2$. The value of $\phi$ was taken from^71^ (i.e., $\phi _n=\phi _h=5$, $\phi _r=2$). The value of $\epsilon$ was considered to be $3\times 10^{-5} \, ms^{-1}$. The $g_{_{GPe->STN}}$ and $I_{app}$ of the STN in the healthy state is $2.2 \, nS/\mu m^2$ and $8.4 \, pA/\mu m^2$, respectively. In the PD state of the network model these two parameters were changed to $7 \, nS/\mu m^2$^72–75^ and $3 \, pA/\mu m^2$^76^, respectively.

To simulate GPe neurons in the network model, we set $\phi _h=0.135$, $\phi _n=0.165$, $\phi _r=1$, and $\epsilon =0.0055$ (similar to^71^). The $g_{_{GPe->GPe}}$, $g_{_{STN->GPe}}$, and $I_{app}$ of GPe in the healthy state were 0.01, $0.01 \, nS/\mu m^2$, and $5.9 \, pA/\mu m^2$, respectively. To simulate the PD state of the network model, these parameters were changed to 0.9, $0.55\, nS/\mu m^2$^72–75^, and $0.5 \, pA/\mu m^2$, respectively. Note that in the PD state of the network model $I_{app}$ of the GPe and GPi decreases leading to less activity of the GPe and GPi neurons due to the increasing striatal inhibition (explained in^56^) in the network model. Parameters of the GPi neurons are similar to the GPe neurons with the difference that for the GPi neurons, $\phi _h=0.1$ and $\phi _n=0.135$. The $g_{_{GPe->GPi}}$, $g_{_{STN->GPi}}$, and $I_{app}$ of GPi in the healthy state were 0.01, $0.005 \, nS/\mu m^2$, and $7.7 \, pA/\mu m^2$. To simulate the PD state of the network model, these parameters similar to GPe neurons were changed to 1.9, $1.1 \, nS/\mu m^2$ , and $4 \, pA/\mu m^2$, respectively. Parameters of the thalamic neurons are the same as in^18^ with the difference that in our network model $g_{_{GPi->Th}}$ was $0.05 \, nS/\mu m^2$. These modifications moved our network model activity more close to the experimental results.

The synaptic model used here is of a conductance-based type similar to the model used in^17,18,61,62,71,77^. The synaptic currents used in Eqs. (1) and (13) are computed as follows:

28 $$\begin{aligned} \mathrm {I_{pre \rightarrow post}} = g_{_{pre \rightarrow post}}(v-E_{pre \rightarrow post})\sum s_j, \end{aligned}$$

where the *j* is the index of the presynaptic neuron. The parameter *s* in Eq. (28) is

29 $$\begin{aligned} \mathrm {s^{'}} = \alpha H_{\infty }(v_{pre} - \theta _{pre})(1-s) - \beta _{pre}s, \end{aligned}$$

where the $H_{\infty }(v)$ as follows:

30 $$\begin{aligned} \mathrm {H_{\infty }(v)} = \frac{1}{1 + e^{-(v - \theta ^H)/\sigma ^H}}. \end{aligned}$$

### Population firing rate

To compute the time resolved population firing rate for each neuronal population in the network model we used 10 milliseconds sliding window and shifted with steps of 1 millisecond over the entire simulation time while for each step we counted the number of spikes for all neurons in the population and converted it to spikes per second (sp/s).

### Sensorimotor and DBS pulses

The sensorimotor and DBS pulses are simulated using the following equation:

31 $$\begin{aligned} \mathrm {I_{pulse}} = A \times H\,\left(\sin \left(\frac{2\pi f t}{1000}\right)\right) \times \left(1 - H\,\left(\sin \left(\frac{2 \pi f \left(t + \delta \right)}{1000}\right)\right)\right) \end{aligned}$$

where *A*, *f*, *t*, and $\delta$ are pulse amplitude, frequency, time (in *ms*), and pulse duration (in *ms*), respectively. The *H*(.) is the Heaviside function. To simulate DBS, we set $f=150 \, Hz$ and $\delta =0.1 \, ms$. Pulse amplitude was varied to inspect its impact on the outcome of DBS (see results and Fig. 4D). Other DBS parameters are adopted from^10,54,66,78–81^. For sensorimotor pulses (i.e., cortico-thalamic input), $A=4.5 \, pA/\mu m^2$, $f=20 \, Hz$, and $\delta =5 \, ms$. we generated a Poisson process with the given rate in Eq. (31) in case of irregular DBS pattern.

### Thalamic fidelity

Thalamic neurons in the network model show four types of responses to the cortico-thalamic input pulse: (1) Correct spike: a single thalamic spike in response to a cortico-thalamic input pulse. (2) Missed spike: refers to the case when there is no thalamic spike in response to a cortico-thalamic input spike. (3) Extra spike: refers to the case when a thalamic neuron shows more than one spike in response to a cortico-thalamic input pulse. (4) Undesired spike: occurs when a thalamic neuron spikes while there is no cortico-thalamic input spike (Supplementary Figure S1). According to the four response types of the thalamic neurons to a single cortical pulse, the thalamic fidelity is computed using the following equation:

32 $$\begin{aligned} \mathrm {thalamus \; fidelity}=1-\frac{N_M + N_E + N_U}{N_{exp}} \end{aligned}$$

where the $N_M$, $N_E$, and $N_U$ are the number of missed spikes, extra spikes, and undesired spikes, respectively. The $N_{exp}$ is the number of expected thalamic spikes due to cortico-thalamic pulses. Since each cortical pulse is given to all thalamic neurons in the network model, we expect to observe that each thalamic neuron, relaying the cortico-thalamic pulse, emits a spike in response to the cortico-thalamic pulse. Therefore, the number of expected thalamic spikes in response to the cortical inputs (i.e., $N_{exp}$) equals the number of thalamic neurons multiplied by the number of cortico-thalamic pulses^18,82^.

### Synchrony index

We used Fano Factor (FF) to measure synchronous spiking activity for each neuronal population in the network model. To compute FF we used the following equation:

33 $$\begin{aligned} \mathrm {FF}=\frac{Var(PFR)}{E(PFR)} \end{aligned}$$

where the *Var*(.) and *E*(.) are the variance and mean of the population firing rate (*PFR*), respectively. Higher FF values represent more synchrony in the spiking activity of a neuronal population in the network model^56,83^.

### Mean power spectral density

The power spectral density (PSD) of the population firing rates was computed using Welch’s method in python 2.7 (i.e., using scipy.signal.welch python package;^84^). The sampling rate and the segment length were set to 1000 Hz and 1000 data points, respectively. Other parameters required for the scipy.signal.welch function were set to the predefined default values (see https://docs.scipy.org/doc/scipy-0.14.0/reference/generated/scipy.signal.welch.html). The mean power spectral density was computed by averaging over 50 simulations.

### Oscillation index

The oscillation index was computed by dividing the area under the curve of a PSD in the $\beta$ frequency range (i.e., between 13 to 30 Hz) by the area under the curve for the whole frequency range (i.e., from 1 to 500 Hz). The frequency at which the PSD is maximized was taken as the frequency of the oscillation.

### Excessive thalamic activity

We assumed that the extra spikes of the thalamic neurons during the resting state of the network model (i.e., when there are no cortico-thalamic sensorimotor pulses) are related to pathological impairment. So, to evaluate it, we measured the mean firing rate of thalamic neurons (in sp/s), over the whole simulation period, during the resting state.

### Simulation

The simulations were implemented in python 2.7. All differential equations were solved using odeint from SciPy library^84^ with $0.05 \, ms$ time resolution (see https://docs.scipy.org/doc/scipy-0.18.1/reference/generated/scipy.integrate.odeint.html). To reduce the simulation time, we performed parallel programming using the python message passing interface (MPI) in cluster computing with 30 core processors (Intel $3.2 \, GHz$). To avoid the initial transient network model responses, we did not consider the first $250 \, ms$ of each simulation in our analysis.

## Results

### The network model captures features of the healthy and PD BG

**Figure 2:**
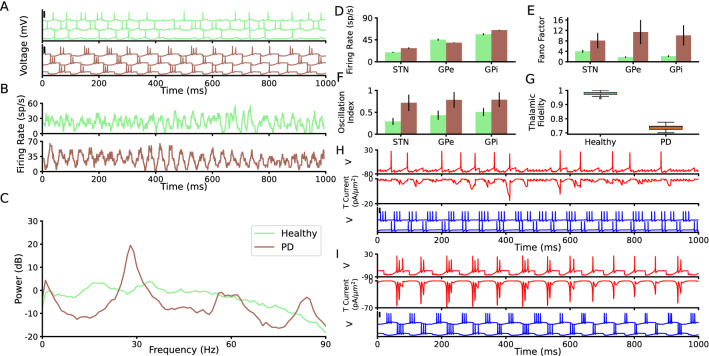
*Neuronal and population properties of BG in healthy and PD states.* (**A**) Membrane potential of three STN neurons in the network model in the healthy state (top) and PD state (bottom). The black vertical thick lines indicate 50 mV. (**B**) Time resolved population firing rate of the STN neurons in the Healthy state (top) and PD state (bottom). (**C**) Mean power spectrum (average of 50 trials) of the STN time resolved population firing rate in the healthy state (light green) and PD state (brown). (**D**–**F**) Population mean firing rate (**D**), Fano factor (**E**), and oscillation index (**F**) of the STN, GPe, and GPi in the network model (error bars show standard deviation; color codes correspond to C). (**G**) thalamic fidelity in the healthy state, PD state, and during regular and irregular EDBS. (**H**) The membrane potential of an STN neuron (top), its corresponding T-type calcium current (middle), and two connected GPe neurons to the STN neuron (bottom) in a healthy state. (**I**) The same as H for PD state.

The activity of different BG regions in the healthy state is non-oscillatory and desynchronized^3,85–87^. This feature is captured in our network model. Similar to the experimental results, the STN spiking activity in the healthy network model is asynchronous irregular (Fig. 2A, top panel; the same for the GPe and GPi spiking activity; Supplementary Figure S2A,D). This is also reflected in the STN population activity in the healthy state of the network model (Fig. 2B, top panel, and also Supplementary Figure S2B,E top panels). The STN population activity in the healthy network model is non-oscillatory. This leads to a flat PSD of the STN population firing rate in the healthy state (Fig. 2C, Supplementary Figure S2C,F). Altogether, these results indicate that the activity of the network model in healthy state is not oscillatory, in accordance with experimental results.

The STN, GPe, and GPi mean population firing rates, in the healthy state of the network model, are $19.4 \pm 1.1 \, sp/s$, $45.47 \pm 1.2 \, sp/s$ and $56.52 \pm 2 \, sp/s$, respectively which match the previously reported experimental values^87,88^.

In the PD state, the network model neurons show synchronized bursts of spiking activities in the $\beta$ frequency range (Fig. 2A,B and also Supplementary Figure S2A,B,D,E, bottom panels). This also matches experimental studies which indicate synchronized $\beta$ band oscillatory spiking activities in the BG as a hallmark of PD^7–10,88–102^.

To bring the network model from the healthy to the PD state, we followed three steps. First, the $I_{app}$ applied to the GPe and to the GPi neurons (Eq. 1) was decreased from $5.9 \, pA/\mu m^2$ and $7.7 \, pA/\mu m^2$ (healthy state) to $0.5 \, pA/\mu m^2$ and $4 \, pA/\mu m^2$(PD state), respectively. This reduction represents the increase in the striato-pallidal inhibition^3,19,103^. This leads to a reduction in the activity of the GPe neurons ($39.1 \pm 0.8 \, sp/s$; independent two-tailed t-test, $p<0.001$) in the PD state of the network model, compared to the healthy state (Fig. 2D). This is in line with the experimental studies indicating that the GPe firing activity decreases during PD^87,104,105^. However, despite decreasing the $I_{app}$ applied to the GPi neurons, the GPi firing rate increases ($64.9 \pm 0.86 \, sp/s$; independent two-tailed t-test, $p<0.001$) compared to the healthy state (Fig. 2D;^104,106,107^). The reason is that the lower GPe activity during the PD state of the network model disinhibits the GPi neurons. Thereby, the GPi population firing rate in the PD network model increases compared to the healthy state (Fig. 2D).

Second, the $I_{app}$ applied to the STN neurons, representing cortico-subthalamic input, in the network model (Eq. 1) was decreased from $8.4 \, pA/\mu m^2$ (healthy state) to $3 \, pA/\mu m^2$ (PD state). Such a change in the network model is in line with the experimental studies showing that the cortical activity decreases in PD^76^ which can lead to less cortico-subthalamic drive, due to direct cortico-subthalamic connectivity^108–110^. Note that despite decreasing the $I_{app}$ of the STN neurons in the PD state of the network model, the STN activity increases ($27.9 \pm 1.5 \, sp/s$; independent two-tailed t-test, $p<0.001$) compared to the healthy state (Fig. 2D)^88,89^. The reason is STN disinhibition due to a reduction in the activity of the GPe units in the PD state of the network model (Fig. 2D).

Third, the synaptic connections in the subthalamo-pallidal circuit (STN to GPe and GPe to STN synapses) were strengthened in the PD state, compared to the healthy state (see Materials and methods). Such subthalamo-pallidal synaptic strengthening is in line with the experimental studies showing that both STN to GPe and GPe to STN synapses are strengthened during PD^72–75^.

Applying these three changes brings the network model from the healthy state to the PD state. STN neurons of the PD network model show synchronized bursts of spiking activity in the $\beta$ frequency range (Fig. 2A; the GPe and GPi neurons show the same behaviour; Supplementary Figure S2A,D). The STN PD-like $\beta$ band oscillations are also observed in the STN population firing rate (Fig. 2B,C) as well as in the GPe and GPi population activities (Fig. 2E,F and also see Supplementary Figure S2).

The pallido-thalamic pathway is important for flowing of information (i.e., the motor information) that is affected by abnormal activity in BG as what has been observed in the previous experimental study^66^. Also, the poor connectivity of the thalamus has been shown in fMRI study in rats with PD^111^. Therefore, we simulated the thalamic information processing (i.e., cortico-thalamic motor commands) by measuring thalamic fidelity (see Materials and method, and see also the Supplementary Figure S1, panels A and B) in the healthy and PD states of the network model. Our results indicate that thalamic fidelity (see materials and methods) to the cortical motor commands decreases in the PD state of the network model compared to the healthy state (Fig. 2G). This result is in line with the previous computational studies^18,59,60,82,112^ indicating a reduction of the thalamic fidelity during PD. This reduction is a results of higher burst rate of the GPi neurons that sent input to the thalamus, compared to the healthy state (See green the Supplementary Figure S1, panels C and D). This observation matches with the previous experimental^54^ and computational studies^61^ and confirms the validity of our results.

The reason behind the generation of the $\beta$ oscillations in the PD state by the model is the rebound bursting activity of the STN neurons due to receiving pallidal inhibition. In line with the experimental studies^91,113,114^, the STN neurons in the network model show rebound bursts after the strong inhibition. When an STN neuron receives the strong burst from a GPe neuron, its T-type calcium current increases to the sufficient value that results in rebound burst of the STN neuron (Fig. 2I). The resulted STN burst then excites the GPe neurons through the excitatory subthalamo-pallidal pathway and reverberates the bursting behaviour. This mechanism leads the $\beta$ oscillations in the population activity. While, in the healthy state, the pallido-subthalamic burst input is not high enough to elicit rebound burst acitivty in the STN neurons (Fig. 2H).

All in all, our network model can reproduce features of the experimental data for both the healthy and PD states of the BG. Mainly, the network model shows asynchronous irregular spiking activity in the healthy state and synchronous $\beta$ band oscillatory activity in the PD state. In addition, thalamic fidelity decreases in the PD state of the network model compared to the healthy state.

### Effects of EDBS and IDBS on the network model

The STN high frequency DBS has therapeutic effects on PD signs such as reducing the pathological $\beta$ oscillations in the cortico-BG loop^33,36,79,115–117^, and improving PD-related motor symptoms^27–32,118–120^. However, the mechanism(s) underlying STN DBS is yet unknown. To understand whether the STN DBS therapeutic effects are due to excitation of STN or inhibition of STN, we applied EDBS (i.e., excitatory DBS) and IDBS (i.e., inhibitory DBS) to the STN neurons in the network model and investigated the effect(s) of each DBS type. We tested which DBS type can quench the PD-like $\beta$ oscillations in the network model. Furthermore, we also tested which DBS type can improve the thalamic fidelity to the cortical motor commands.

**Figure 3:**
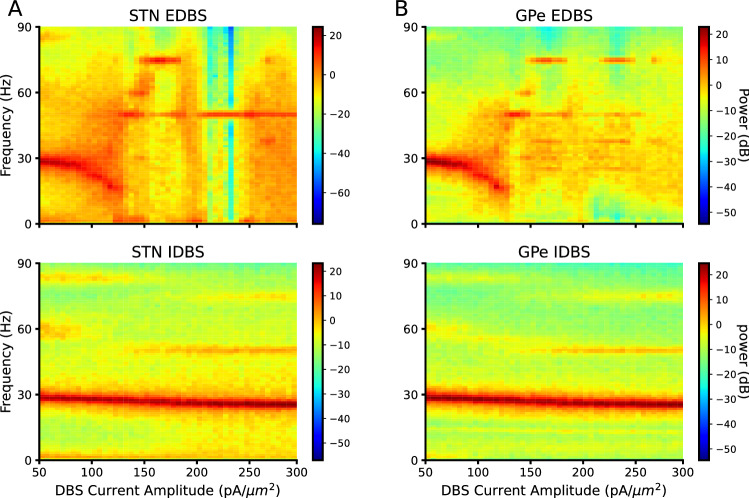
*Spectrogram of STN and GPe in EDBS and IDBS states.* (**A**) The spectrogram of STN across DBS current amplitude in EDBS (top) and IDBS (bottom) states. (**B**) The same as A for GPe. Each point in all spectrograms is averaged over 20 trials.

To this end, we applied various current amplitudes in both types of DBS to STN to see their effect on $\beta$ oscillations. We found that the EDBS with high enough amplitude (approximately 120 pA; Figure 3 and Supplementary Figure S3A,B, top panels) can quench the $\beta$ oscillations in STN and GPe populations, while the IDBS failed to quench the $\beta$ oscillations in all applied current amplitudes (Fig. 3 and Supplementary Figure S3A,B bottom panels).

**Figure 4:**
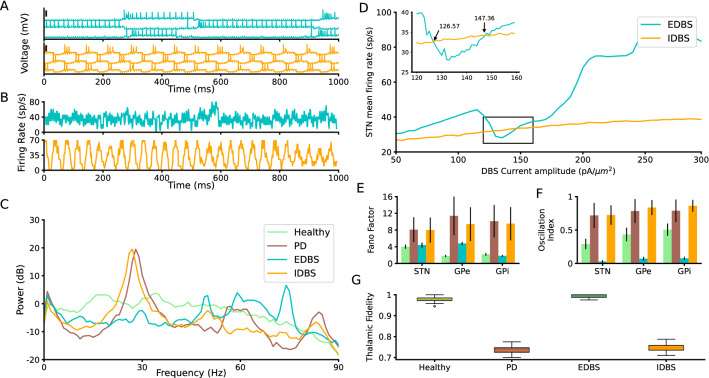
*Neuronal and population properties of BG in EDBS and IDBS states.* (**A**) Membrane potential of three STN neurons in the network model in the EDBS (top) and IDBS (bottom). The black vertical thick lines indicate 50 mV. (**B**) Time resolved population firing rate of the STN neurons in the EDBS (top) and IDBS (bottom). (**C)**Mean power spectrum (average of 50 trials) of the STN time resolved population firing rate in the healthy state (light green), PD state (brown), and during EDBS (cyan) and IDBS (orange). (**D**) The STN mean firing rates for IDBS and EDBS is shown versus the amplitude of the stimulation current. Inset is the zoomed-in presentation of the results in the rectangle. Each point in the plots is averaged over 20 trials. The IDBS curve crosses the EDBS curve at 126.57 and 147.36 pA points. (**E** and **F**) Fano factor (**E**), and oscillation index (**F**) of the STN, GPe, and GPi in the network model (error bars show standard deviation; color codes correspond to C). (**G**) thalamic fidelity in the healthy state, PD state, and during regular and irregular IDBS.

For a better comparison of two DBS types, we chose the amplitude of DBS such that the STN mean firing rate is approximately the same for two DBS types. To this end, we computed the STN mean firing rate (over 20 trials) by varying $A = 50 \, pA/\mu m^2$ to $300 \, pA/\mu m^2$ (Fig. 4D), then we applied the constraint that the firing rate of the STN in excitatory DBS (EDBS) and inhibitory DBS (IDBS) to be equal. Therefore, to simulate EDBS, we set $A=126.57 \, pA/\mu m^2$, $147.36 \, pA/\mu m^2$, and $f=150 \, Hz$. Parameter settings for IDBS were the same as EDBS, with the difference that $A=-126.57 \, pA/\mu m^2$ and $-147.36 \, pA/\mu m^2$. Due to the similarity of results, we reported the DBSs with $A=147.36 pA$ except for the Fig. 6.

#### EDBS and IDBS effects

Applying the EDBS (see materials and methods) to the STN neurons in the network model quenches the PD-like $\beta$ oscillations in the STN (and in the other regions included in the network model; Fig. 4A–C, top panels).

**Figure 5:**
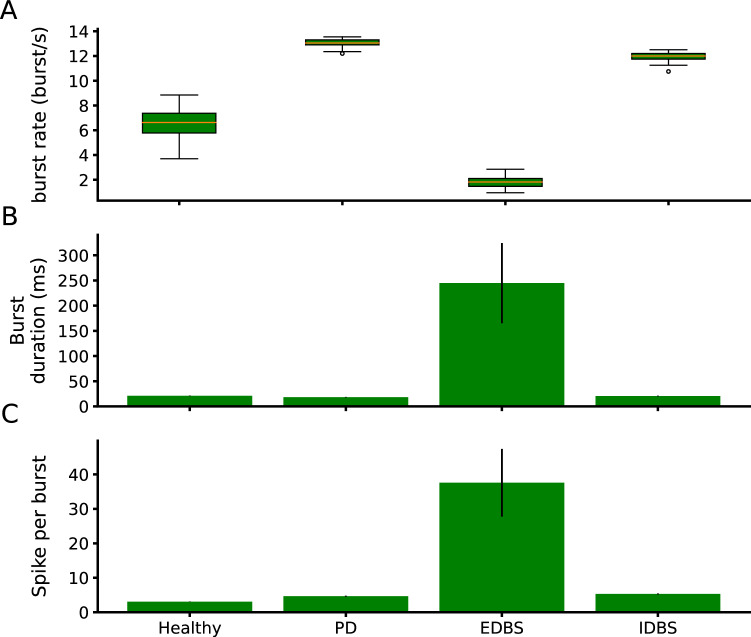
*Burst profile of GPi neurons.* (**A**) Burst rate of GPi neurons in healthy, PD, regular EDBS, irregular EDBS, regular IDBS and irregular IDBS states. (**B** and **C**) Mean burst duration (in msec) and mean the number of spikes per burst in healthy, PD, regular/irregular EDBS, and regular/irregular IDBS states. The error bars show standard deviation.

To quantify the performance of EDBS on the PD-like $\beta$ oscillations in the network model, we measured the Fano factor and the oscillation index of the STN, GPe, and GPi population firing rates while the STN was stimulated. For EDBS, both Fano factor and oscillation index dramatically decreased compared to the PD state ($P < 0.001$, independent two-tailed t-test), for all regions measured (i.e., STN, GPe, and GPi; Fig. 4E,F). We also compared the performance of the EDBS on improving the thalamic fidelity in the network model. We found that EDBS increased the thalamic fidelity (almost to the healthy level; Fig. 4G). In addition, we found that the burst rate of the GPi neurons significantly decreased ($P<0.001$, independent two-tailed t-test) compared to the PD and also to the healthy (Fig. 5A). It matches the previous experimental ^54^ and computational studies^61^ and indicates the validity of our model.

This result indicates that applying regular/irregular EDBS to the STN neurons in the network model, quenches the PD-like $\beta$ oscillations and improves the thalamic fidelity to the cortical motor command.

Next, we tested whether high-frequency inhibition of the STN neurons (IDBS) has the same effects as the EDBS in the network model. To this end, we stimulated the STN neurons in the pathological network model by high-frequency inhibitory pulses (see materials and methods) and measured the PSD, Fano factor, and oscillation index of the STN population firing rate as well as the thalamic fidelity in the network model (Fig. 4). Our simulation results show that IDBS (unlike the EDBS) fails to quench PD-like $\beta$ oscillations and does not improve the thalamic fidelity (Fig. 4). Although the burst rate of the GPi neurons in regular and irregular IDBS slightly decreases ($p<0.001$, independent two-tailed t-test) compared to the PD state, the GPi burst rate was still above the healthy state when the network model exposed to IDBS (Fig. 5A). However, comparing the performance of EDBS and IDBS in the network model reveals that EDBS outperforms IDBS in improving PD signs (i.e., quenching PD-like $\beta$ oscillations and improving the thalamic fidelity) in the network model. To check whether irregular IDBS (see Materials and methods) can quench PD signs, we applied both types of DBS with irregular pattern to STN model network. The results were similar to the regular DBS case (see Supplementary Figure S4). The results were the same as the regular manner of the DBS.

#### IDBS can not treat the pathological STN rebound bursting activity in the network model

**Figure 6:**
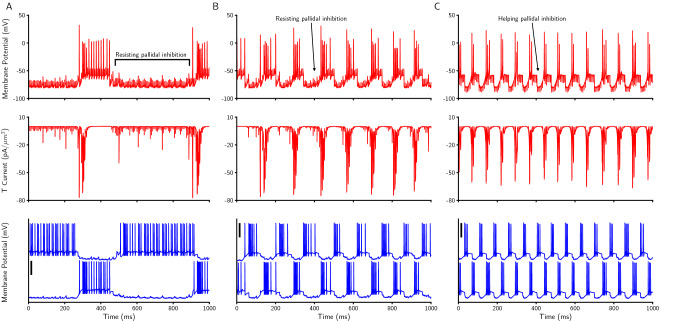
*The role of inhibitory rebound bursting in STN neurons during EDBS and IDBS.* (**A**) Membrane potential of an STN neuron (top), its corresponding T-type calcium current (middle; see materials and methods), and two connected GPe neurons to the STN neuron during EDBS with the current of 147.36*pA*. (**B**) The same as A, EDBS with the current of 126.57*pA*. (C) The same as A, during IDBS. The black vertical thick lines indicate 50 mV.

We showed that only EDBS (and not IDBS) is able to quench the PD-like $\beta$ oscillations in the network model (Fig. 4). The reason why IDBS fails to quench the PD-like $\beta$ oscillations in the network model is the rebound bursting activity of the STN neurons due to IDBS. In line with the experimental studies^91,113,114^, the STN neurons in the network model show rebound bursts of spiking activity after the strong inhibition. As the IDBS is a barrage of inhibitory inputs to the STN neurons in the network model (see Materials and methods), the STN neurons react to it by rebound bursts of spiking activities (Fig. 6C). STN rebound bursts lead to an increase in the GPe spiking activity through subthalamo-pallidal excitatory pathway. Then, the higher GPe activity gives rise to inhibition of the STN neurons which results rebound bursting due to the T-type calcium current (Fig. 6C). Such a mechanism retains the PD-like $\beta$ oscillations in the network model during IDBS. Therefore, the reason why IDBS fails to quench the PD-like $\beta$ oscillations in the network model is the propagation of the STN rebound bursts through the subthalamo-pallidal loop.

During EDBS, two behaviours can occur depending on the current amplitude. First, for 147.36*pA*, the STN neurons in the network model do not show rebound bursting activity (Figs. 4, and 6A). The reason is that the STN spiking activity is driven by the EDBS pulses, counteracts with the pallido-subthalamic inhibition (see also Fig. 2I), thereby no STN rebound bursting activity can happen due to the non-sufficient T-type calcium current. In other words, EDBS reduces rebound excitation of STN via T-type calcium currents, due to depolarization of STN neurons. Second, for 126.57*pA*, the EDBS extends the burst durations and the inter-burst intervals which results quenching the PD-like $\beta$ oscillations (Fig. 6B).

### Excessive thalamic activity in resting state

#### Excessive thalamic activity occurs only in PD

**Figure 7:**
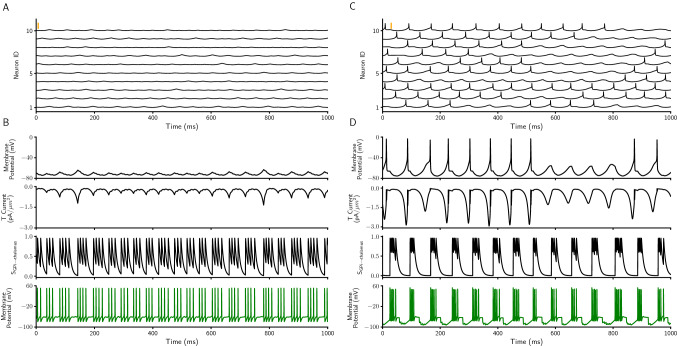
*Excessive thalamic activity profile in healthy and PD state.* (**A**) Membrane potential of 10 thalamic neurons in the healthy network model during resting state. The orange vertical thick line indicates 50 mV. (**B**) From top to bottom, membrane potential, low threshold T-type calcium current, synaptic inhibitory input from the connected GPi and membrane potential of the corresponding GPi neuron (see Materials and methods) in the healthy state of the network model. (**C**,**D**) The same as A and B for PD state of the network model.

**Figure 8:**
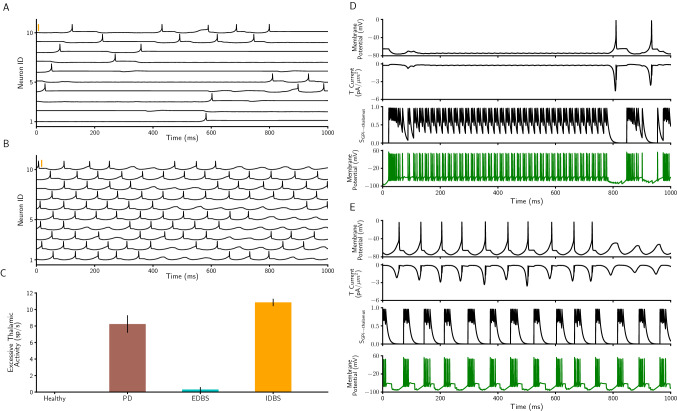
*Excessive thalamic activity profile of PD state during EDBS and IDBS.* (**A**,**B**) The membrane potential of 10 thalamic neurons when STN is exposed to regular EDBS (A) and regular IDBS (**B**) during the resting state of the network model. The orange vertical thick lines indicate 50 mV. (**C**) Excessive thalamic spike rate in the healthy and PD states and when the STN is exposed to EDBS and IDBS (error bars show standard deviation). (**D**) From top to bottom, membrane potential, low threshold T-type calcium current, synaptic inhibitory input from the connected GPi and membrane potential of the corresponding GPi neuron (see Materials and methods) when STN is exposed to EDBS in the network model. (**E**) The same as D, when STN is exposed to the IDBS.

So far, using our simulation results, we showed that the STN EDBS (and not IDBS) can quench pathological $\beta$ oscillations emerging in the PD state of the network model. Next, we investigated the effect(s) of each stimulation type (i.e., EDBS or IDBS) on the resting state activity of the healthy and the PD network model. To simulate the resting state of the network model, we removed the cortico-thalamic sensorimotor pulses (i.e., by setting the $I_{SMC}$ in the Eq. (13) to zero for both healthy and PD network model simulations; see materials and methods). Our simulation results indicate that the thalamic neurons in the healthy network model, do not show any spiking activity during the resting state (Fig. 7A). However, in the PD network model, the thalamic neurons show the mean spiking activity of $8.24 \pm 1 \, sp/s$ during the resting state (Fig. 8C and also see Fig. 7C).

The thalamic neurons in the network model receive inhibitory inputs only from the GPi neurons (Fig. 1A; note that the cortico-thalamic input is set to zero during the resting state). Thereby, the resting state thalamic activity in the network model is driven by inhibitory inputs from the GPi. Our resting state simulation results show that the healthy GPi spiking activity is tonic (Fig. 7B, bottom panel). Such tonic GPi spiking activity or weak bursting activity (i.e. lower spikes per burst; Fig. 7B, two bottom panels; and also see Fig. 5B,C) do not sufficiently increase the T-type calcium current of the thalamic neurons. This leads to no thalamic spiking activity during the resting state and in the healthy condition of the network model (Fig. 7B, top panel). However, in the PD condition, the strong bursting activity of GPi sufficiently increases the T-type calcium current of thalamic neurons which leads them to excessive spikes (Fig. 7D).

#### STN EDBS suppresses the excessive thalamic activity in the network model

Next, we investigated the effect(s) of EDBS and IDBS on the excessive thalamic activity in the network model. Mainly, we tested which DBS type (i.e., EDBS or IDBS) can reduce the resting state excessive thalamic spiking activity in the PD network model. Our simulation results show that applying EDBS to the STN neurons in the network model leads to better performance as compared to applying the IDBS (Fig. 8). In other words, STN EDBS reduces the excessive thalamic spiking activity and brings it back almost to the healthy state while STN IDBS fails to do so (Fig. 8A–C ). In the following, we explain why STN EDBS outperforms STN IDBS in improving the excessive thalamic activity in the network model.

In line with previous experimental and computational studies^54,61^, GPi neurons in the PD state of our network model switch between long-lasting hyperactivity and no activity states when the STN is stimulated by the EDBS (Fig. 8D, bottom panel and also see the burst duration and number of spikes per burst in Fig. 5B,C). During the GPi hyperactivity periods, thalamic neurons are strongly inhibited and thereby do not show excessive spiking activity anymore (Fig. 8D). However, a single thalamic spike occurs at the end of the GPi activity (Fig. 8D, top panel; also see Fig. 8A). This single thalamic spike is due to the after-hyperpolarization increase in the T-type calcium current of the thalamic neuron receiving the GPi strong inhibitory input (Fig. 8D).

However, when the STN is exposed to the IDBS, the GPi neurons in the network model show regular oscillatory bursts of spiking activity in (Fig. 8E, bottom panel). These strong GPi bursts (similar to PD state; see Fig. 5A) cause sufficient flow of the T-type calcium current of the thalamic neurons in the network model (see the T current fluctuations in Fig. 8E) which consequently, leads to excessive thalamic spiking activity. Our simulations show that these results do not depend on the temporal pattern of the stimulation and hold also for irregular DBS. We investigated the thalamic activity when the STN exposed to both types of DBS with irregular pulses and the results show the same effect as the regular pulses (Supplementary Figure S4G).

Altogether, our simulation results indicate that the EDBS outperforms the IDBS, not only in quenching the PD-like $\beta$ oscillations in the network model (Fig. 4), also in reducing the resting state excessive spiking activity of the thalamic neurons nearly to the healthy state (Fig. 8C).

## Discussion

Permanent and excessive $\beta$ oscillations in BG is the hallmark of the PD^11–13^. The DBS quenches $\beta$ oscillations and improves motor symptoms related to PD^27–33,36,79,115–120^. But, the mechanism of the DBS is poorly understood. In the present study, we investigated the generation of the pathological $\beta$ oscillations in the BG by neural modelling and the effects of the DBS in different scenarios. We showed that the subthalamo-pallidal circuit is the potential source of the generation of the $\beta$ oscillations based on the computational network model. Our findings confirm that the rebound burst activity of the STN neurons is the key reason for to generation of the $\beta$ oscillations as was suggested in^17,23^. Then we investigated the role of the DBS in quenching the $\beta$ oscillations. We applied inhibitory and excitatory DBS on STN neurons to compare their effect with those observed in experimental studies. Thereby, we tuned the current amplitude of each DBS types to have equal STN firing rates. The results show that the EDBS can counteract the pallido-subthalamic inhibition and therefore, can suppress the rebound burst activity of the STN neurons (Fig. 6). Also, EDBS can extend the duration of STN bursts which results in quenching the $\beta$ oscillations (Fig. 6). however, the IDBS is not able to quench the $\beta$ oscillations due to its ineffectiveness in the suppression of rebound burst activity of STN neurons and the extension of burst duration. Our results suggest that the effect of DBS on STN neurons is excitatory. Our simulation results also help to clarify the relationship between tremor and the activity of the BG and thalamus. We showed that the postinhibitory rebound activity of the thalamic neurons due to the strong inhibition of the GPi neurons is the potential reason for the resting state tremor. Like other signs of PD, the tremor activity of the thalamus was quenched by EDBS while the IDBS worsened the tremor activity.

In the following, we discuss the network model, the generation of $\beta$ oscillations, the tremor, and the role of DBS in improving the PD signs in detail.

### Network model

Our BG model has been created using the Hodgkin-Huxley type neuron model that can generate neural behaviour in detail (i.e., ion currents). Previous computational BG studies^17,18,57–62,77,121^ except^24,122^ which were created by Hodgkin-Huxley type neurons, were not reported $\beta$ oscillations in PD state. In these studies, one of the changes to set the model in PD state is strengthening subthalamo-pallidal synapses, based on the experimental results^72–75^. The PD $\beta$ oscillations also were reported by the model proposed in^56^ which was created using the LIF neuron model. This model moves to the PD state only by increasing striato-pallidal spiking activity (without changing any synaptic strength). They show the PD $\beta$ oscillations might arise from the network effects, without taking into account the details of neuronal dynamics. While ^23^ used integrate-and-fire-or-burst neuron in their network model for subthalamo-pallidal circuit. Their model moves to PD state by changes in synaptic strength and input spikes in subthalamo-pallidal circuit and represents the PD $\beta$ oscillations. In this model, the rebound burst activity of the STN and GPe neurons played an important role in the generation of the $\beta$ oscillations. The PD $\beta$ oscillations have also reported in the firing rate models^20,123–125^. These models cannot be used for single neuron study in the network. By contrast, the role of calcium bursting can not be demonstrated in these models. However, the modified BG model in the present study was able to generate the $\beta$ oscillations in the PD state. Also, the other properties of the BG model (i.e., firing rates and changes in firing rates when moving from the healthy to the PD state) were matched the experimental observations. these modifications were made by changing some intrinsic and connectivity neuronal properties (see Materials and methods).

### Generation of beta oscillations

Rhythmic activity is a widespread dynamics of the brain circuits. While normal oscillations in healthy brain are crucial for transmission of the information between the brain regions^126–130^, abnormal synchrony disrupts efficient communication and is a hallmark of several neurological and cognitive disorders (epilepsy, PD, schizophrenia, etc). For example, enhanced $\beta$ oscillations observed in the cortex and several nuclei of BG is a common trait of the brain dynamics in PD^6,7,9,10^. However the source of these oscillations is still under debate. Computational and experimental studies have implicated the subthalamo-pallidal circuit as the potential source of the $\beta$ oscillations in PD^7,8,14–24,82^. Moreover, the induction of $\beta$ oscillations from the cortex into the BG has been claimed by^6,25,26^. On the other hand, the role of striatum and the pallido-striatal loop in the generation of beta oscillations have been demonstrated^63–65^. Our study is based on the assumption that the subthalamo-pallidal loop is the main source of the $\beta$ oscillations. As we did not modeled the striatum, it was not possible to check the role of this population in the pathological $\beta$ oscillations in this study and the results may be different if the striatal population is added to the model.

More detailed models for the generation of the $\beta$ oscillations, the rebound burst activity of the STN neurons due to the inhibition of the GPe neurons in the BG has been considered^15,16,114^. Somehow, this hypothesis is confirmed when the motor symptom of patients with PD suppressed after receiving a T-type calcium channel blocker such as Zonisamide^131^. Also, the computational studies such as^17,18,23,24,122^ confirmed this hypothesis. While, the other studies demonstrate that the excessive inhibition on inhibitory population (such as striato-pallidal inhibition) and/or excessive excitation on excitatory population (such as cortico-subthalamic) result in $\beta$ oscillations^20,56,77,132,133^. Our findings confirm the role of post-inhibitory rebound bursts of STN neurons in the generation of the $\beta$ oscillations (Fig. 2H,I).

### The role of the DBS is excitatory

The DBS improves PD-related motor symptoms^27–32,118–120^ and PD-related neuronal behaviour in BG^10–13,33,36,79,115–117^. In our network model, we quantified the PD-related signs by $\beta$ oscillations, synchrony, thalamic fidelity, and tremor frequency (see below, materials and methods, and also Fig. 2). Here, we applied two types of the DBS in our network model to see which satisfy our expectation about the amelioration of PD signs with DBS. Our findings suggest that the EDBS improved the PD neuronal behaviour and the motor symptoms while the IDBS worsened them (Figs. 3 and 4). Only a small shift of $\beta$ peak in PSD of the STN was observed after application of IDBS (Fig. 4C). This shift may come from the change in burst rate and burst duration of STN due to extra inhibition despite the pallidal inhibition.

Although the previous studies^38–41,43–47,49,50,134^ indicate that the role of the DBS on its target is inhibitory, in^48,51,53–55^ has been demonstrated the opposite role by our findings. On the other hand, the computational study in^56^ has claimed the excitation of excitatory and/or inhibition of inhibitory populations leads the network to oscillations (in this case is $\beta$ oscillations). Therefore, the pathological $\beta$ oscillations have been quenched using high-frequency inhibitory spike trains on STN, which is not consistent with EDBS in our network. Still, there is opposite evidence for this study’s results: the initiation of movement accompanied with increasing cortical activity^135–138^ and the STN neurons are excited by cortex^108–110^ which quenches briefly the $\beta$ oscillations in patients with PD^8,139,140^ and a computational model proposed in^24^. Moreover, the neuronal bursting activity was not investigated in that study, though in^141^ a computational model was proposed based on^56^ that the neuronal bursting activities have been investigated. This study showed the variety of STN burst range can affect $\beta$ oscillations. In detail, the low burst rate of STN neurons quenched the $\beta$ oscillations while the high STN burst rate generated the $\beta$ oscillations with a little shift in frequency. Indeed, the intrinsic behaviour of STN neurons in a non-pathological state is bursty and the higher burst rate of BG nuclei in PD condition is also shown^142^ (and see also Fig. 5 for GPi) which is consistent with the results reported in^141^. The consistency of this study and our network model is justified with considering that 1-the STN burst rate in our network model corresponds to high STN burst rate of the model in^141^ and 2-with considering the STN burst rate when it exposes to EDBS corresponds to low STN burst rate in that study. Consequently, it seems to the therapeutic effects of DBS acts by excitation of the STN.

However, it has been recently shown that the GPe orchestrates the $\beta$ rhythm in the mice BG using the optogenetic method^65^. It is shown that either inhibition or excitation of STN does not decrease the pathological $\beta$ oscillations in the network while the pallidal inhibition does. Also, the $\beta$ induction in STN neurons using optogenetic excitation does not generate the $\beta$ oscillations in cortical activity, while the optogenetically induction of $\beta$ in the GPe generates the $\beta$ oscillations in ECoG. Inconsistency of these observations with our results may be rooted in the fact that continuous optogenetically excitation/inhibition may have different effects from those of high-frequency electrical pulses. For instance, BG nuclei are partially affected by optogenetic stimulation, while, electrical DBS affects all the neurons in the nucleus, and supposedly parts of neighboring nuclei^143^. Besides, the inhibition of the GPe results in the reduction of subthalamic inhibition which abolishes the STN post-inhibitory rebound bursts. Also, induction of the $\beta$ rhythm in GPe by optogenetical excitation results in STN inhibition that causes STN rebound burst. These observations confirm the role of the STN rebound bursts in the generation of $\beta$ oscillations in our study.

On the other hand, in^37,144,145^ it is shown that the DBS inhibits the STN neurons while it induces spike in their axons. In this hypothesis, despite the inhibition of STN neurons, their post-synaptic neurons (GPe and GPi) receive excitatory neurotransmitter (glutamate)^55^. So, inducing excitatory pulses in STN neurons in the network model matches this hypothesis. Our results suggested that the post inhibitory rebound burst of the STN neurons is the main cause of the $\beta$ oscillations (Fig. 2I). The EDBS by counteracting the pallidal inhibition in the network model eliminates the rebound bursting of the STN neurons, while the IDBS enhances the pallidal inhibition resulting in more rebound burst activity of the STN neurons in the network model (Fig. 6).

Altogether, our results showed that when the Parkinsonian $\beta$ oscillation are generated by STN-GPe network, only excitatory stimulation of the network can weaken the $\beta$ oscillations. The results were not limited to the regular high frequency stimulations and with irregular pattern of DBS pulses, the same effects have been observed (Supplementary Figure S4). However, in^146,147^ it is shown that both regular and irregular pattern of the DBS fail to quench pathological oscillations. We observed this effect in our model when using different DBS parameters (i.e. DBS amplitude current)^148^. In this study, we did not observe significant changes in pathological oscillations when the STN exposed to the irregular DBS.

### Excessive thalamic activity in resting state

The tremor in patients with PD occurs when they are at rest. The frequency of the resting tremor in PD state is reported 3 to 8 Hz^69,92,149–152^ and it correlates with thalamic neuronal activity^153^. Previous computational studies^17,18,57^ demonstrated that the tremor is highly correlated with single neuron spiking activity in the tremor frequency in BG populations such as STN. While in^92^ the correlation between the high-frequency activity of STN and tremor has been shown. In a computational study, the tremor activity in the PD state has been represented by synaptic input from the GPi to the thalamic neurons in tremor frequency^62^. However, the resting state was not simulated in mentioned computational studies. In our network model, we simulated the resting state with interrupting sensorimotor command pulses to the thalamic neurons. Our results, show the extra thalamic spikes in tremor frequency in the PD state (and not in the healthy state).

In previous experimental studies it is shown that the rhythmic bursting activity of some of the thalamic cell is correlated with tremor^68,69^. They called these thalamic neurons the tremor cells. The excessive thalamic activity in our network model did not match the rhythmic bursting activity with tremor cell. Moreover, with applying DBS in our network model the frequency of the excessive thalamic activity were decreased, while the previous behavioural studies have reported that by applying STN DBS the frequency of limb activity increased and while the amplitude of the limb movement was suppressed^81^. Therefore we hypothesize that the thalamic neurons in our model do not represent the thalamic tremor cells. Meanwhile, as the frequency of resting state excessive thalamic activities in our network model matches the tremor frequency and occur only in PD state, they might be related to tremor.

The resting state excessive thalamic activities were quenched when the network model exposed to the EDBS, while these activities were increased when the network model exposed to the IDBS. This finding, again, confirmed that the role of the DBS is excitatory.

## Conclusion

We utilized a computational model of the BG to investigate the underlying mechanisms of the DBS. With the help of the model we concluded that first, the rebound burst activity related to the T-type calcium current of the neurons has a key role in the generation of $\beta$ oscillations. Second, we found that the role of the STN DBS is excitatory (and not inhibitory), because, the excitation of the STN neurons suppressed their rebound burst. Third, again, the rebound burst of thalamic neurons gave rise to the generation of resting tremor. Next, by exposing the STN neuron to high-frequency excitation the excessive thalamic activities-which may be related to tremor were quenched, while, exposing the STN neurons to high-frequency inhibition has been worsened. In summary, based on our model, we conclude that the role of the high-frequency DBS is excitatory on its target.

## Supplementary Information

Supplementary Information.
