# Supplementary material for: Excitatory deep brain stimulation quenches beta oscillations arising in a computational model of the subthalamo-pallidal loop
Source: Sci Rep. 2022 May 12;12:7845. doi: 10.1038/s41598-022-10084-4 (PMC9098470; doi:10.1038/s41598-022-10084-4)
Supplement: Supplementary file 1 — Supplementary Information. [file 41598_2022_10084_MOESM1_ESM.pdf]

Supplemental Figures

Excitatory Deep Brain Stimulation Quenches Beta Oscillations Arising in a Computational Model of the Subthalamo-Pallidal Loop

Seyed Mojtaba Alavi, Amin Mirzaei, Alireza Valizadeh, Reza Ebrahimpour*

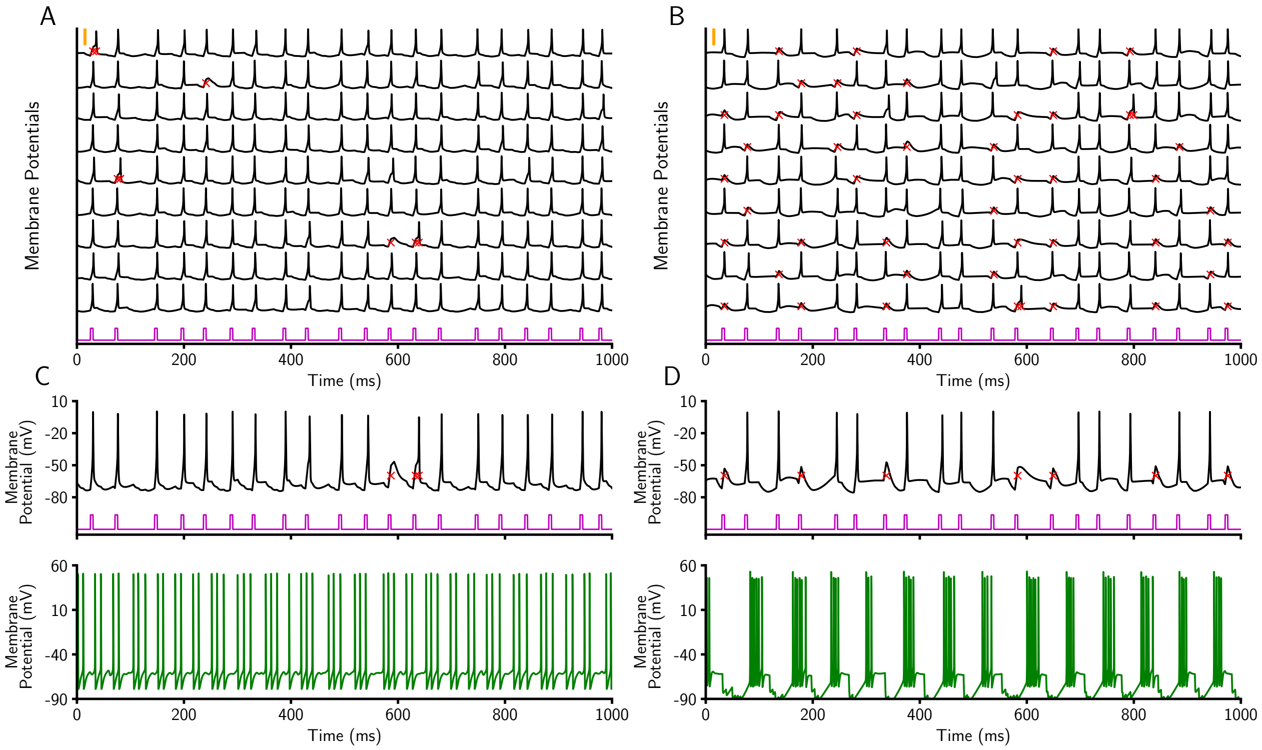

Supplementary figure S 1. Thalamic response to cortical inputs. (A) Membrane potential of 10 thalamic neurons in the healthy state. The purple signal is the cortical input to each thalamic neuron. The red crosses indicate wrong or missed response to the cortical input. (B) The same as A for PD state. The orange vertical thick lines indicate 50 mV. (C) Membrane potential of one sample of thalamic neuron from A (top) and the membrane potential of a connected GPi neuron (bottom) in healthy state. (D) The same as C in PD state.

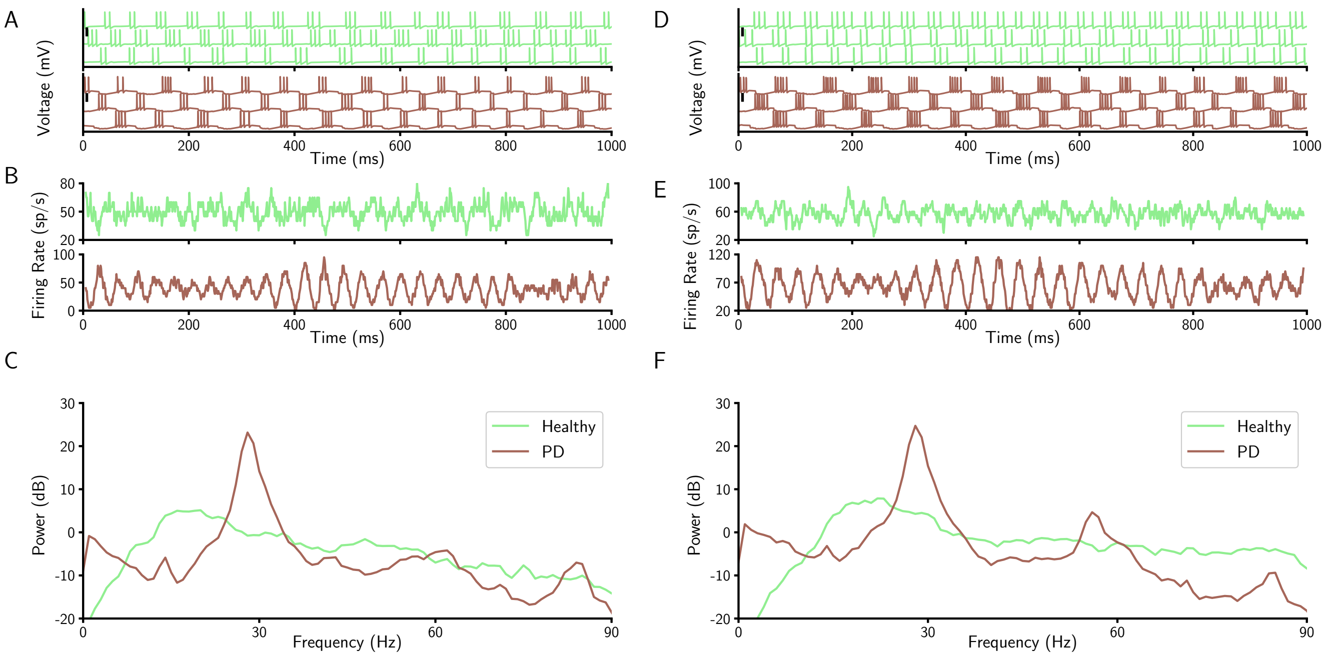

Supplementary figure S 2. Neuronal and population properties of GPe and GPi in the healthy and PD states. (A) Membrane potential of three GPe neurons in the network model in the healthy (top) and PD (bottom). The black vertical thick lines indicate 50 mV. (B) Time resolved population firing rate of the GPe neurons in the healthy (top) and PD (bottom). (C) Mean power spectrum (average of 50 trials) of the GPe time resolved population firing rate in the healthy state (light green), PD state (brown). (D-F) The same as A-C for the GPi.

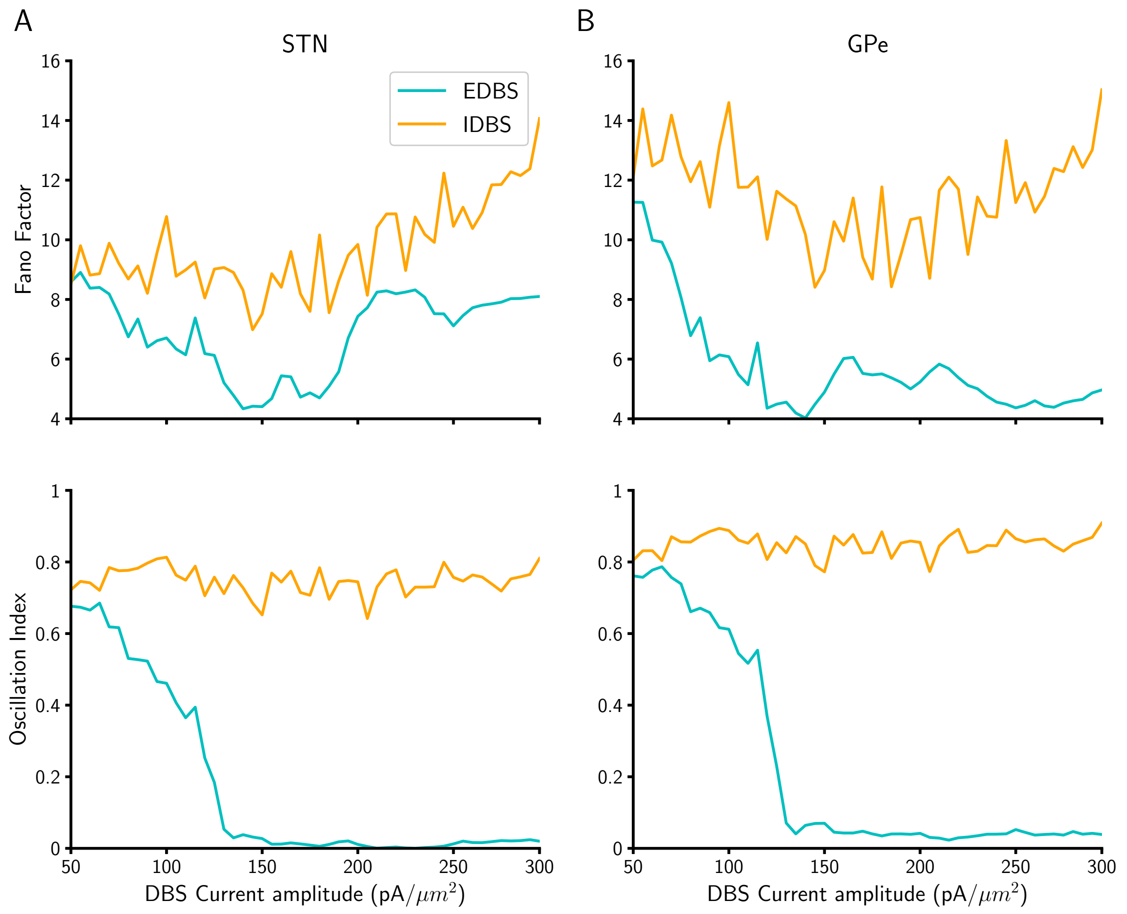

Supplementary figure S 3. Fano factor and Oscillation index of STN and GPe across various DBS current amplitudes. (A) The fano factor (top) and oscillation index (bottom) of STN across various DBS currents amplitudes during EDBS (cyan) and IDBS (orange). (B) The same as A for GPe. Each point in all panels is averaged over 20 trials.

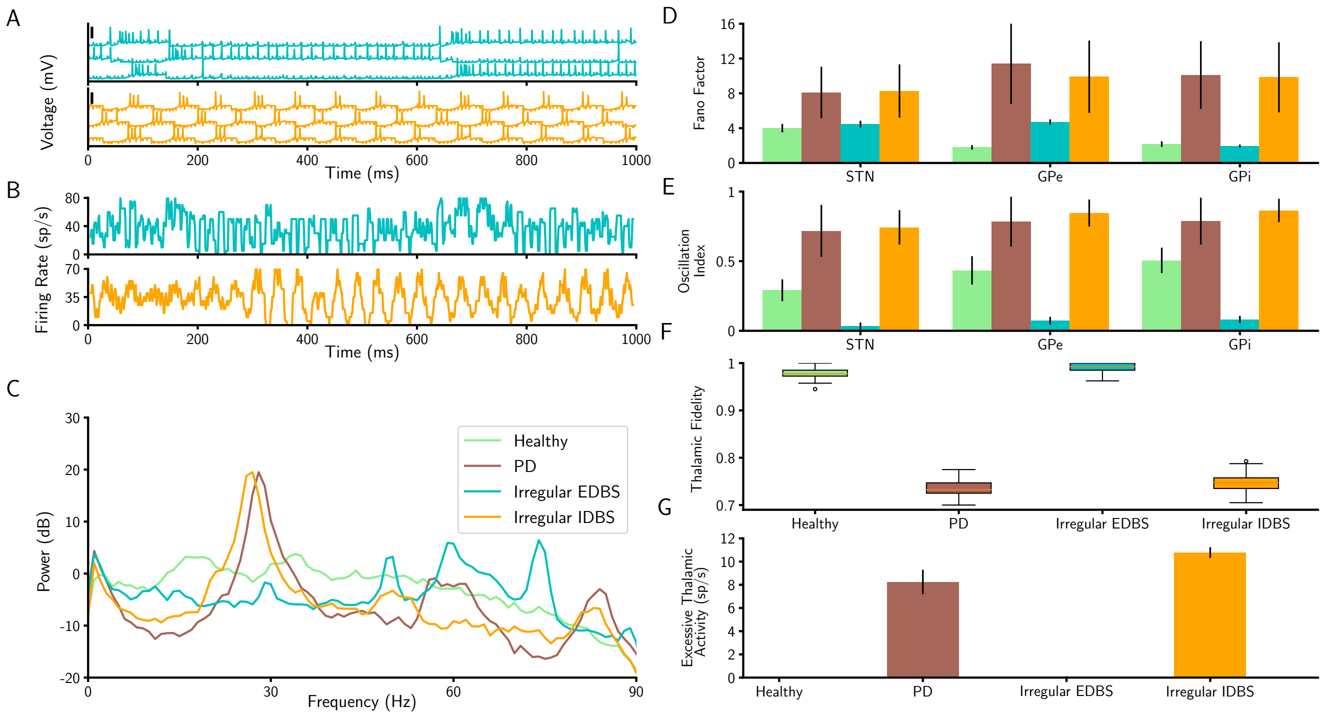

Supplementary figure S 4. Neuronal and population properties of BG in Irregular EDBS and IDBS states. (A) Membrane potential of three STN neurons in the network model in the irregular EDBS (top) and irregular IDBS (bottom). The black vertical thick lines indicate 50 mV. (B) Time resolved population firing rate of the STN neurons in the irregular EDBS (top) and irregular IDBS (bottom). (C) Mean power spectrum (average of 50 trials) of the STN time resolved population firing rate in the healthy state (light green), PD state (brown), and during irregular EDBS (cyan) and irregular IDBS (orange). (D and E) Fano factor (D), and oscillation index (E) of the STN, GPe, and GPi in the network model (error bars show standard deviation; color codes correspond to C). (F) thalamic fidelity in the healthy state, PD state, and during regular and irregular IDBS. (G) Tremor-like frequency of thalamus in the healthy and PD states and when the STN is exposed to irregular EDBS and irregular IDBS (error bars show standard deviation).
